# Generation of Sub-nanosecond H Atom Pulses for Scattering from Single-Crystal Epitaxial Graphene

**DOI:** 10.1021/acs.jpca.2c05364

**Published:** 2022-10-16

**Authors:** Kai Golibrzuch, Victoria Walpole, Anna-Maria Schönemann, Alec M. Wodtke

**Affiliations:** Max-Planck-Institute for Multidisciplinary Sciences, Am Faßberg 11, and Institute for Physical Chemistry, Georg-August-University Göttingen, Tammannstrasse 6, D-37077Göttingen, Germany

## Abstract

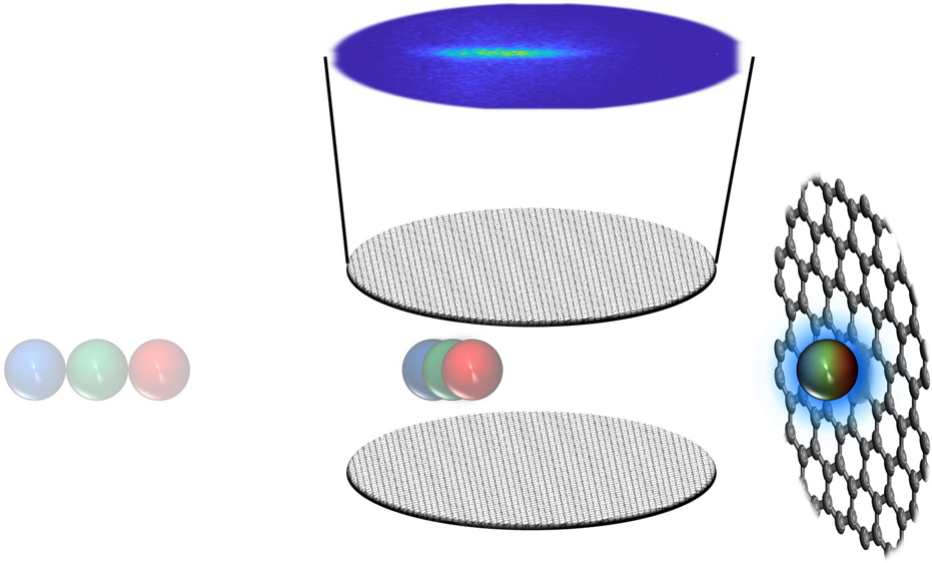

Pulsed molecular beams allow high-density gas samples to be cooled to low internal temperatures and to produce narrow speed distributions. They are particularly useful in combination with pulsed-laser-based detection schemes and have also been used as pump pulses in pump–probe experiments with neutral matter. The mechanical response of pulsed valves and chopper wheels limits the duration of these pulses typically to about 10–100 μs. Bunch compression photolysis has been proposed as a means to produce atomic pulses shorter than 1 ns—an experimental capability that would allow new measurements to be made on chemical systems. This technique employs a spatially chirped femtosecond duration photolysis pulse that produced an ensemble of H atom photoproducts that rebunches into a short pulse downstream. To date, this technique could not produce strong enough beams to allow new experiments to be carried out. In this paper, we report production of pulsed H atom beams consistent with a 700 ps pulse duration and with sufficient intensity to carry out differentially resolved inelastic H scattering experiments from a graphene surface. We observe surprisingly narrow angular distributions for H atoms incident normal to the surface. At low incidence energies quasi-elastic scattering dominates, and at high incidence energy we observe a strongly inelastic scattering channel. These results provide the basis for future experiments where the H atoms synchronously collide with a pulsed-laser-excited surface.

## 1 Introduction

While dynamics of so-called half-collisions^1^ can be observed in the time domain via photodissociation pump–probe methods with time resolution on the order of femtoseconds,^2,3^ current methods to temporally resolve full collisions fail to achieve better than a few microseconds resolution.^4^ This contrast in performance arises from the obvious and dramatic differences associated with manipulating light compared to mechanical objects. The shortest pulsed beams of neutral matter are produced using HI photolysis; for example, it was possible to produce H atom pulses shorter than 1 μs by photodissociation of a molecular beam of HI.^5−7^ Here the product of the characteristic length scale of the photodissociation volume and the H atom velocity limits how short the pulse may be. Such beams of H atoms have been used to perform scattering experiments from polycrystalline graphene grown on Pt(111),^8,9^ for which there is now a large body of important work.^10−17^ We know for example that at low normal incidence energies the scattered H atoms exhibited quasi-elastic scattering. With an increasing normal component of the translational energy, a second inelastic scattering channel was seen and attributed to transient C–H bond formation. These observations were consistent with a barrier to chemisorption of ∼0.4 eV.

The aim of this work is to demonstrate successful H atom scattering experiments from graphene using much shorter H atom pulses than in prior work. This would mark an important step toward synchronized scattering experiments where H atoms interact with a laser excited surface that has not had time to return to equilibrium. Photolysis of HI(*v* = 0) using spatially chirped femtosecond laser pulses was recently shown to produce much shorter (5–7 ns) H atom pulses. The idea here is to utilize the inherent wavelength spread of an ultrafast laser pulse to spread out the laser light in space so that higher energy photons dissociate HI molecules farther from the target than do lower energy photons. Hence, the H atom photoproducts formed with more translational energy have further to fly to the target. If done correctly, the H atom ensemble bunches together at the target. A proof-of-concept experiment was reported in 2014.^18^ Unfortunately, the H atom pulses produced in that work were far too weak to be used in a scattering experiment.

In this work, we show results from a new apparatus especially designed for surface scattering experiments with ultrashort intense H atom pulses. The key improvements include the use of femtosecond UV laser pulses with pulse energies up to 20 mJ and ion imaging with resonance-enhanced multiphoton ionization (REMPI) for detection of scattered atoms. The generated atom pulses have a maximum observed pulse duration of ∼3 ns, limited by the temporal resolution of the detection scheme. Using a model of the experiment to describe the intensity distribution of the ionizing laser beam and its pointing instability, we find that the H atom pulses likely exhibit a pulse duration of about 700 ps. We demonstrate the application of these ultrashort H atom pulses in scattering experiments from single-crystal epitaxial graphene (EG) grown on Ir(111). The energy and angle resolved data exhibit features of quasi-elastic and strongly inelastic scattering transient chemical bond formation. Both channels exhibit very narrow angular distributions.

## 2 Experimental Setup

**Figure 1:**
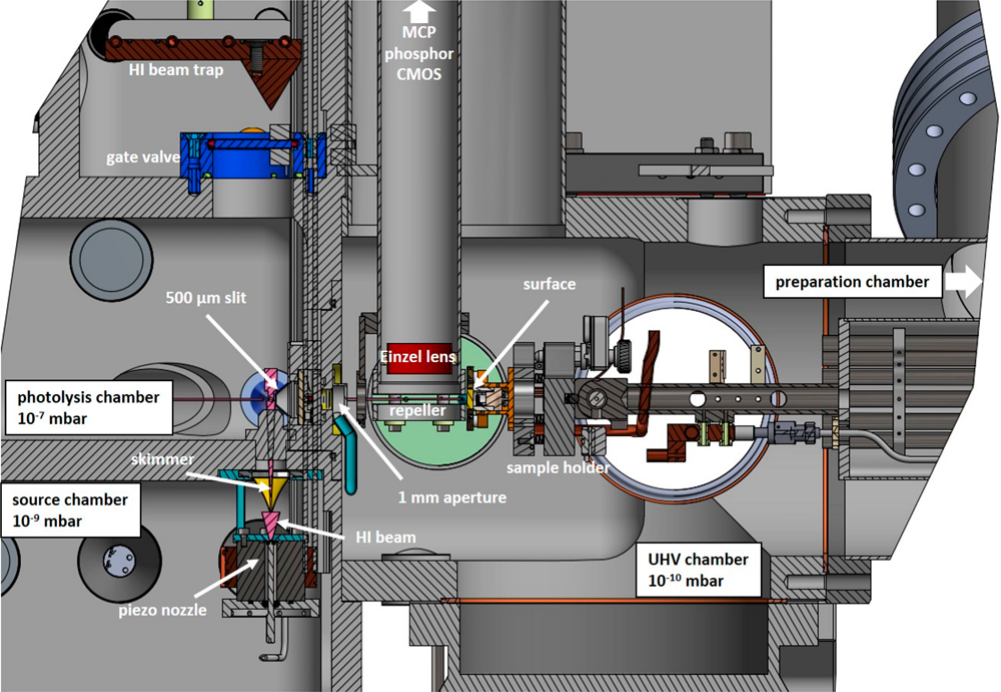
H atom scattering apparatus. The apparatus consist of three vacuum chambers connected by small apertures. The source chamber holds a piezo-driven pulsed valve, emitting a molecular beam of HI seeded in argon (pink). The HI beam passes a skimmer (yellow) and enters the photolysis chamber. A photolysis laser dissociates a part of the HI beam. The H atoms pass through a 500 μm slit and a 1 mm round aperture before entering the ultrahigh-vacuum chamber, which contains the surface and ion imaging detector. Indicated pressures are base pressures of the respective vacuum chambers.

A schematic of the experimental apparatus is shown in Figure 1. It consists of a source, a photolysis, and a UHV scattering chamber, separated from one another by differential pumping apertures. The source chamber is pumped by a 1500 L/s cryo-pump (Leybold, COOLVAC 1500iCL) and is equipped with a home-built pulsed piezoelectric actuated valve^19−21^ operating at 50 Hz, from which a 3 bar mixture of 10–20% hydrogen iodide (HI) diluted in argon expands to 5 × 10^–4^ mbar. The resulting molecular beam pulses are 40 μs long and contain ∼4 × 10^16^ HI molecules rotationally cooled to ∼14 K. About 0.04% of these HI molecules pass through a 1 mm diameter skimmer located 2 cm in front of the nozzle orifice before entering the photolysis chamber, which is pumped by a 300 L/s turbomolecular pump (TMP) (Pfeiffer Vacuum, HiPace 300P) to 1 × 10^–6^ mbar. The photolysis chamber employs active N_2_ sealing gas flow with a base pressure of 1 × 10^–8^ mbar to protect the bearing at the high-pressure side of TMP against corrosion. The HI beam with a density of ∼10^15^ HI cm^–3^ intersects the photolysis laser beam 75 mm downstream, where a small fraction (∼2%) of the HI is photodissociated. H atom photoproducts are emitted in an angular distribution peaking at 90° with respect to the molecular beam direction, some of which pass a slit (5 mm × 0.5 mm, parallel to molecular beam direction) and then a 1 mm circular aperture before entering the main ultrahigh-vacuum (UHV) chamber. The residual molecular beam strikes a liquid nitrogen cooled beam trap to reduce HI pressure in the photolysis chamber and to avoid exposure of the TMP to corrosive HI. The cold trap can be separated from the photolysis chamber via an internal gate valve for HI recovery.

The UHV chamber operates at 4 × 10^–10^ mbar (base pressure 2 × 10^–10^ mbar) and is pumped by a 700 L/s TMP (Pfeiffer Vacuum, HiPace 700) backed by a second 100 L/s TMP (Pfeiffer Vacuum, HiPace 90). It is sealed to the photolysis chamber by a home-built slide valve, which allows venting of the photolysis and source chambers while maintaining UHV. The UHV chamber comprises a scattering and a preparation subchamber. The preparation subchamber is equipped with an ion sputter source (STAIB, IG-5-C), Auger electron spectrometer (STAIB, ESA 100), low-energy electron diffraction (LEED, OCI Vacuum Microengeneering Inc.), and a residual gas analyzer (SRS, RGA200). The surface is positioned on a four-axis manipulator (Metallic Flex) mounted in line with the H atom beam axis. The sample can be rotated 90° for sputtering, annealing, Auger, and LEED measurements. It is further mounted to a mirror mount-like assembly (see Figure 1) controlled by piezoelectric motors (Physik Instrumente, PiezoMike N-470.11U). This allows an extremely accurate adjustment of the incidence angle of the H atom beam onto the sample, which may limit the H atom pulse duration at the surface. Overall, the orientation of the manipulator in combination with the piezo-controlled tilt angle allows a full 6D control of the sample orientation. Electron bombardment heating allows surface temperatures up to 1500 K, limited by the type K thermocouples attached to the sample.

**Figure 2:**
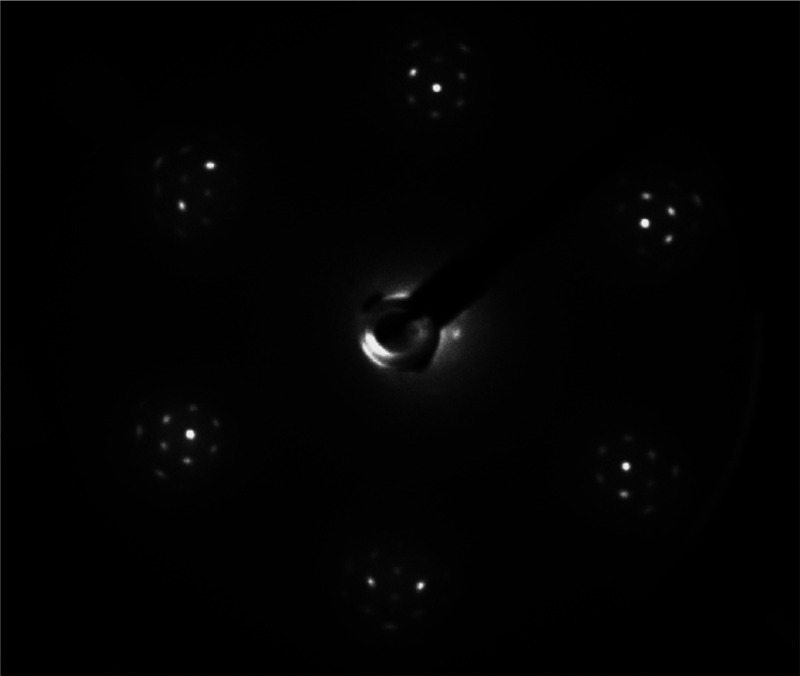
LEED pattern of single-crystal epitaxial graphene grown on Ir(111). The 6-fold symmetry indicates single crystallinity. The closely spaced spots arise from the Moiré pattern formed between the graphene and the iridium.

Epitaxial graphene was grown on an Ir(111) single crystal. Prior to the graphene synthesis, the Ir(111) is cleaned by several cycles of Ar^+^ sputtering and annealed to 1173K in 4 × 10^–7^ mbar oxygen to remove carbon impurities from the surface and the bulk. Finally, several flash-annealing cycles to 1400 K under UHV conditions recover the (111) surface structure. The cleanliness and structure of the Ir(11) surface were checked by Auger spectroscopy and LEED, respectively. The cleaned and annealed Ir(111) crystal was then kept at 1273K and exposed to 1.6 × 10^–7^ mbar of ethylene for 10 min (72 ML) leading to formation of a full graphene monolayer. Figure 2 shows a typical LEED pattern of the EG/Ir(111) sample, exhibiting the signs of a Moiré pattern,^22^ proving graphene is formed in a single rotational domain.

The scattering subchamber is equipped with differentially pumped optical windows and houses an ion imaging detector similar to that used in earlier work by Harding et al.^23^ It consists of a 40 mm repeller grid electrode, a 40 mm extractor grid electrode, and a 495 mm stainless steel tube that acts as a field-free region. A single Einzel lens at the bottom of the flight tube allows for velocity map imaging operation. Ions are amplified on a 56 mm Chevron microchannel plate (MCP) detector (TOPAG), and the resulting electrons are accelerated onto a P43 phosphor screen (ProxiVision). The ion spots are imaged on a CMOS camera (LaVision, M-Lite 2M) by a *f*/0.95 50 mm focal length lens. The MCP detector is time-gated by a home-built high-voltage pulse generator for *m*/*z* selection. The rather high velocity of the generated H atoms requires a high extraction voltage on the repeller grid—typically 4 kV—to map the H^+^ ions onto the MCP detector.

Velocity mapping of H^+^ ions of scattered H atoms was achieved by ionizing H atoms with ns-REMPI at a 5 mm distance to the surface. To reach a high signal intensity, it is advantageous to move the detection laser close to the surface as a large range of scattering angles, and final velocities can be ionized at once. However, ions must fly into the volume where velocity mapping is optimal, located 20 mm from the surface. If this distance is too large, the ion cloud becomes too large to be accurately analyzed with velocity map imaging. On the other hand, if the surface–REMPI distance is too large, the measurement suffers from low scattering signal and significant background. We found that a surface–REMPI distance of 5 mm is a good compromise to obtain angular resolved velocity distributions within a reasonable measurement time.

**Figure 3:**
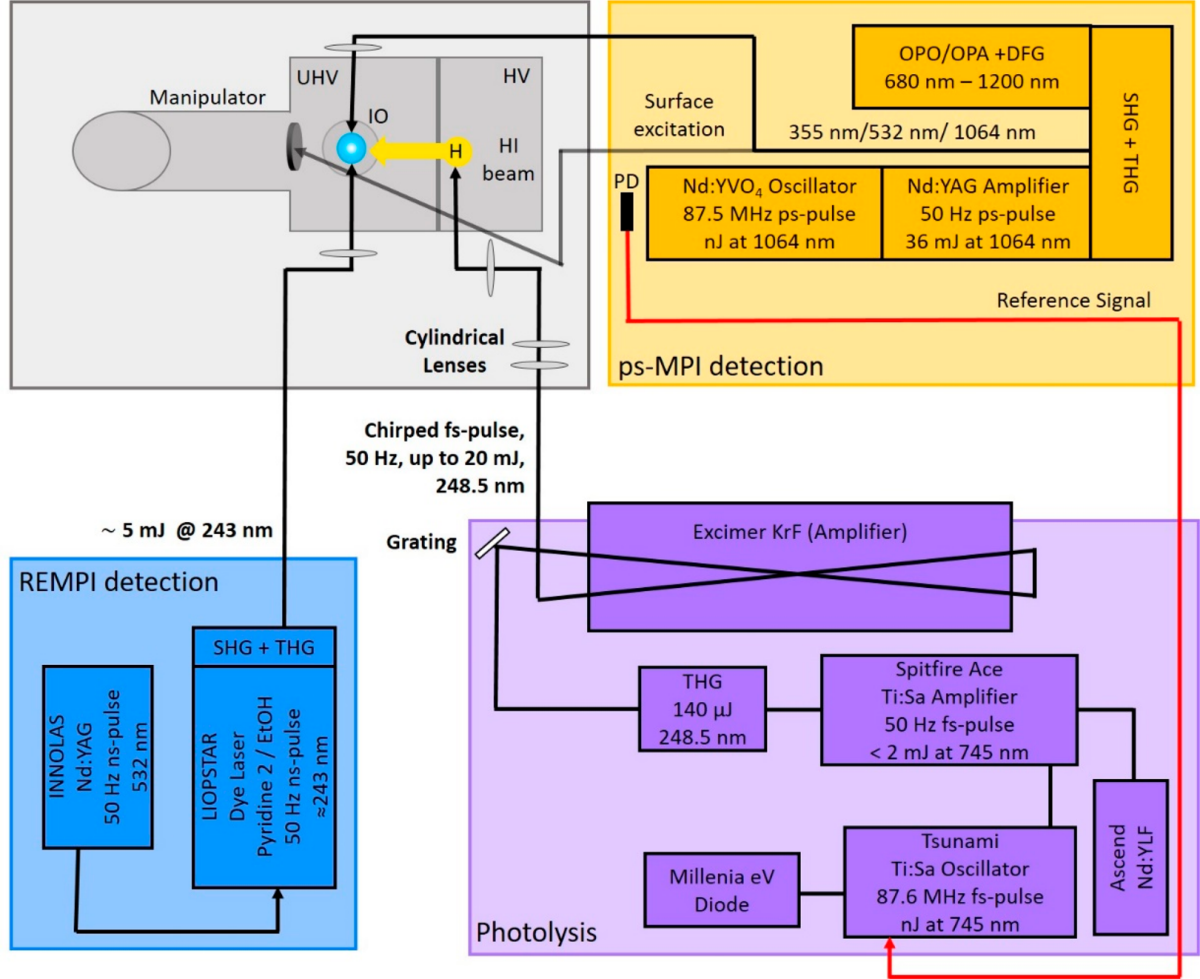
Schematic layout of the overall experiment. Laser systems are shown as violet (photolysis), blue (ns REMPI detection), and yellow (ps-MPI detection). The vacuum apparatus is drawn in gray schematically and shown in greater detail in Figure 1.

Short hydrogen atom pulses are produced with bunch compression photolysis using a specially designed laser source.^18^ The laser system provides ultrashort laser pulses at 248.5 nm with pulse energies up to 20 mJ at 50 Hz repetition rate (violet box in Figure 3). The laser system consists of a Ti:Sa oscillator (Spectra-Physics, Tsunami, 87.6 MHz, 500 mW) seeding a regenerative amplifier (Spectra-Physics, Spitfire-Ace, 50 Hz, 2 mJ, 745 nm, 120 fs, *M*^2^ < 1.3). Third-harmonic generation (Spectra-Physics, THU) of the Ti:Sa output results in 248.5 nm UV laser pulses with a typical pulse energy of 140 μJ. The UV pulses are then directed onto a diffraction grating to generate the angular dispersion necessary for bunch compression,^18^ before being amplified by passing twice through a KrF excimer amplifier (Institute for Nanophotonics eV, LLG-PRO 10). The concept of short-pulse amplification in excimer discharges has been described earlier.^24−27^ The excimer amplifier has an optimized electrode design to increase the width of the discharge and is equipped with a low-jitter thyratron and drift compensating electronics. The discharge is sealed by two rectangular CaF_2_ windows.

We emphasize the advantage of placing the diffraction grating before the final amplification step. Typical blazed UV diffraction gratings have an efficiency of ∼50–60% for the first-order diffraction. Because the KrF amplifier acts as a low signal amplifier, a loss in the seed beam intensity does not affect the output intensity significantly, and we easily achieve gain-saturated pulse energies of up to 20 mJ. The amplified photolysis beam passes a cylindrical telescope (diverging), which increases the beam divergence perpendicular to the H atom beam direction, and is finally focused into the photolysis region by a lens doublet to reduce spherical aberrations consisting of a *f* = 1000 mm and *f* = 750 mm fused silica lens (387 mm effective focal length at 248.5 nm).

The implementation of a diverging cylindrical telescope increases the vertical size of the laser beam in the photolysis region along the molecular beam direction, thereby increasing the photolysis volume. This has several advantages: (1) reduction of saturation effects from the high intensity photolysis laser pulses, (2) reduction of unwanted nonresonant HI ionization, and (3) increase in photolysis volume. The latter increases the H atom beam intensity by a factor of 4–8 without influencing the observable pulse duration.

For detection of incident and scattered H atoms, we used (2 + 1) resonance-enhanced multiphoton ionization via the 2s(^2^S_1/2_) ← 1s(^2^S_1/2_) transition at ∼243.1 nm. The nanosecond pulses are produced from a frequency-tripled dye laser (LIOPTEC LiopStar, Pyridin 2 in ethanol, 4 mJ) pumped by the second harmonic of a 50 Hz Nd:YAG laser (Innolas, SpitLight 600–50, 320 mJ, 6–7 ns) (blue box in Figure 3). The REMPI laser is focused at the center of the ion-imaging system between the repeller and extractor grids, using a *f* = 300 mm fused silica lens. The detection laser focus can be translated in the H atom beam direction by moving the lens with a translational stage. The laser pulse duration of the nanosecond REMPI laser system limits its temporal resolution for H atom measurements to about 7 ns.

We implemented nonresonant multiphoton ionization (MPI) from a picosecond laser source (Ekspla PL2231, 50 Hz, 25 ps, *M*^2^ < 2.5) (orange box in Figure 3) to achieve an improved time resolution for H atom pulse duration measurements. This laser consists of a mode-locked Nd:YVO_4_ oscillator (87.6 MHz, nJ) followed by a regenerative amplifier and a dual pass power amplifier to yield pulse energies up to 35 mJ at 1064 nm. Third-harmonic generation produces 355 nm pulses with a pulse energy of up to 9 mJ, which allows efficient nonresonant MPI detection (ps-MPI).

A part of the Nd:YVO_4_ oscillator beam is coupled into a single-mode fiber and serves as a synchronization signal for the Ti:Sa oscillator of the photolysis laser system. The delay between the photolysis and MPI laser can be varied in two steps. The Ti:Sa laser provides an external trigger for the ps-MPI laser amplifiers; i.e., the trigger pulse selects a respective oscillator pulse to be amplified. This sets the delay between the two laser pulses within the oscillator period time of 11.4 ns. Further adjustment of the delay between the two laser pulses is accomplished by phase shifting (Spectra-Physics, model 3931) the MPI laser synchronization signal provided to the Ti:Sa oscillator. During pulse duration measurements, we use the phase shifting to scan the delay between the two laser pulses and measure the absolute time delay on fast oscilloscope (LeCroy WaveSurfer 510, 1 GHz, 10 GSa/s) using two fast photodiode signals. The photodiode signals are fitted with Gaussian functions to determine the timing. We estimate the time accuracy limit of this method to about ±20 ps.

Bunch compression photolysis has been described in detail before,^18^ and we review the concepts as they specifically apply to this work. The KrF gain medium restricts the photolysis wavelength to 248.5 nm, allowing two H atom kinetic energies (velocities) of 0.99 eV (13.78 km/s) and 1.94 eV (19.25 km/s) as HI exhibits two dissociation channels to form I*(^2^P_1/2_) or I(^2^P_3/2_).^5−7^

The dispersion  at a central laser frequency *v*_0_ needed for ideal bunch compression is linear and defined by eq 1
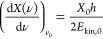
1where *X*_0_ is the distance at which the H atoms are optimally bunched, *h* is the Planck constant and *E*_kin,__0_ is the kinetic energy of the H atoms produced by photolysis at *v*_0_. The spatial frequency distribution produced by the diffraction grating and the focusing lens is given by eq 2
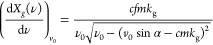
2where *f* is the focal length of the focusing optics, *k*_g_ is the grating period in lines/mm, *m* is the diffraction order, and *a* is the incidence angle of the photolysis laser onto the diffraction grating. *X*_0_ and *E*_kin,__0_ determine the ideal linear dispersion (eq 1) for bunch compression. The grating’s spatial frequency distribution (eq 2) needs to match this value.

In practice, a reasonable combination of diffraction grating and focusing lens is selected, and the incidence angle is optimized to fulfill the bunch compression conditions at the experimental geometry. Table 1 summarizes the conditions used in our setup using a 248.5 nm photolysis laser, and *X*_0_ = 97 mm. Using the same focal length lens, bunch compression of the *E*_I_ = 0.99 eV dissociation requires a 2400 lines/mm grating, whereas the fast *E*_I_ = 1.92 eV channel uses a 1200 lines/mm grating. Consequently, the photolysis volume for the slower dissociation channel is approximately 2 times larger.

**Table 1:** Parameters for Bunch Compression Photolysis of HI at a Central Wavelength of 248.5 nm and a Bunch Compression Distance of 97 mm^a^

	H + I	H + I*
*E*_I_ (eV)	1.94	0.99
*v*_I_ (km/s)	19.25	13.78
grating (lines/mm)	1200	2400
incidence angle (deg)	36	53
diffraction order	1	1
lens focal length (mm)	400	400

a All parameters are given of dissociation of HI(*v* = 0, *J* = 0). Higher rotational and vibrational states produce faster hydrogen atoms.

## 3 Results

**Figure 4:**
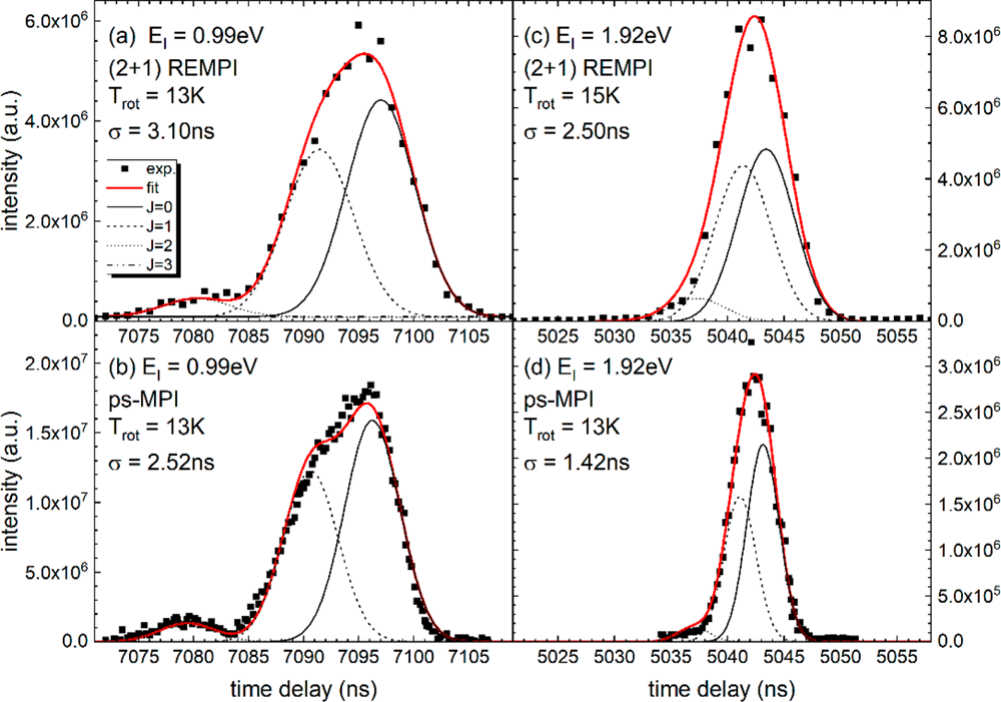
Observed bunch-compressed H atom pulses. Panels a and c employed 5–7 ns laser pulses for (2 + 1) REMPI while panels b and d employed 25 ps laser pulses for nonresonant MPI. A 3 bar expansion of 20% HI seeded in argon was photodissociated. The left column (a, b) shows the H + I* channel while the right column (c, d) shows the H + I channel.

Figure 4 shows measurements (black squares) of the H atom pulses using both ns 2 + 1 REMPI (upper row) and ps-MPI (lower row). The left column shows results for the H + I* channel, and the right column show the H + I channel. In all examples, the pulse is composed of subpulses originating from different ro-vibrational quantum states present in the HI beam. Equation 3 describes a theoretically expected arrival time distribution of H atoms originating from photolysis of HI
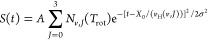
3where the H atom velocity *v*_H_(*v*,*J*) depends on the initial HI quantum state populated in the HI beam and *N*_*v*,*J*_(*T*_rot_) is a Boltzmann quantum state population distribution at temperature *T*_rot_. The velocity *v*_H_(*v*,*J*) of the incident H atoms is calculated from spectroscopic constants for HI and the photolysis laser frequency. Each contributing quantum state is assigned a single pulse width σ. When *A*, σ, *T*_rot_, and *X*_0_ are optimized to fit the experiment, the red lines in Figure 4 are obtained, with *T*_rot_ = 13 K. For analysis of the ps-MPI data (Figure 4b,d), the derived values of σ are found to be 2.52 and 1.42 ns for *E*_I_ = 0.99 eV and *E*_I_ = 1.92 eV, respectively. For ns REMPI (Figure 4a,c) these values are influenced by the detection laser pulse duration and are larger. H atoms originating from HI(*v* = 0, *J* = 0) and HI(*v* = 0, *J* = 1) are not resolved, but H atoms from photolysis of HI in *v* = 0, *J* = 2 can be clearly seen for the H + I* channel.

We next show that the size of the ionizing laser’s focus limits the temporal resolution of the detection. To achieve this, we performed numerical simulations to characterize the laser focusing conditions in the ps-MPI experiments using measured laser beam parameters and the experimental geometry. We generate random start and detection points to define individual H atoms trajectories and calculate the respective flight times, which are then time binned to simulate the experimentally measured H atom pulse profile. The start and detection points are generated with a probability distribution reflecting a Gaussian laser focus intensity distribution, represented by eqs 4 and 5:
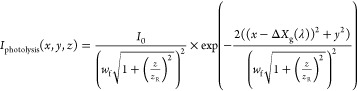
4
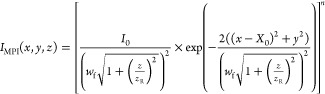
5Here, the *z*-axis defines the laser propagation direction while photolysis and ps-MPI laser are separated by the bunch compression distance, *X*_0_ (= 97 mm) along the *x*-axis. The shape of the laser beam focus depends on its Rayleigh length *z*_R_ and beam waist *w*_f_.
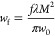
6
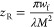
7We account for the nonideal laser focus using the beam quality factor *M*^2^. For a diffraction-limited Gaussian beam, *M*^2^ = 1. The spatial chirp is described by
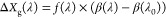
8
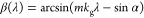
9where β(λ) is the wavelength-dependent grating diffraction angle and *f*(λ) is the wavelength-dependent focal length of the photolysis lens setup. *k*_g_ and *α* can be found in Table 1. Table 2 lists the laser beam and geometry parameters used in the simulation.

**Table 2:** Parameters Used in Numerical Simulation of H Atom Bunch Compression Photolysis

	parameter	value
photolysis laser	*f*_photolysis_ (248.5 nm)	387.95 mm
	λ_0_	285.5 nm
	FWHM	1 nm
	*w*_0_	12 mm
	*M*^2^	1.3
	*w*_f_	3.07 μm
	*z*_R_	99.2 μm
	simulation range	*x* = −1, ..., 1 mm
		*y* = −0.1, ..., 0.1 mm
		*z* = −1, ..., 0.1 mm
ps-MPI laser	*f*_MPI_	250 mm
	λ	355 nm
	*w*_0_	2.4 mm
	*M*^2^	2.5
	*w*_f_	29.4 μm
	*z*_R_	3.06 mm
	*n*	1
	simulation range	*x* = −0.1, ..., 0.1 mm
		*y* = −0.1, ..., 0.1 mm
		*z* = −3, ..., 0.3 mm

We then construct a grid of photolysis and detection points with a grid spacing of 100 nm. We create random points within the grids of both photolysis and detection volumes using the *randsample* function of MatLab R2020a. The probability for each random choice is given by the laser intensity distributions (eqs 4 and 5). We calculate the vector from the respective photolysis position to the MPI detection position and include it only if that trajectory passes through the apertures in the experiment.

**Figure 5:**
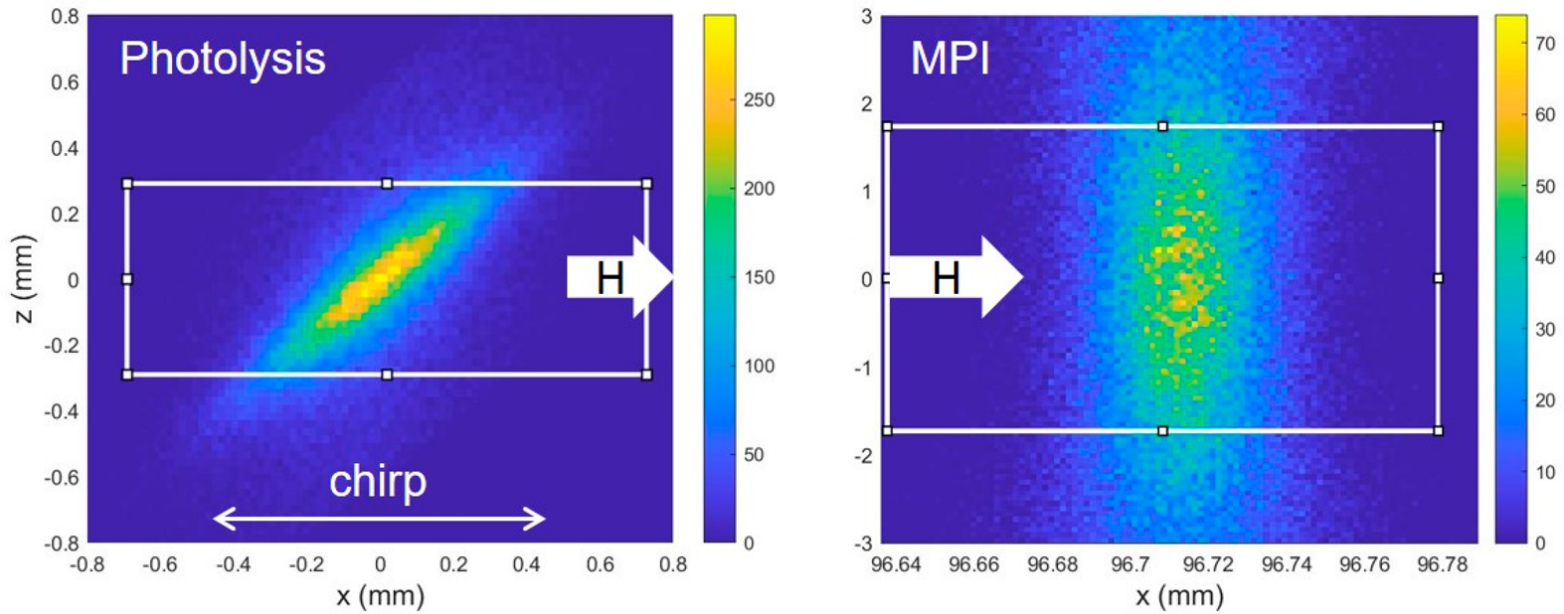
*x*- and *z*-spatial distributions of photolysis (left) and detection points (right) used in the numerical simulation of the H atom bunch compression photolysis. The white rectangles show the regions where trajectories are physically able to pass through the experimental apertures. Results are shown for the bunch compression conditions for 1.92 eV H atoms. +*x* is the H atom propagation direction, and +*z* is the laser propagation direction.

Figure 5 shows a histogram of the distribution of 10^5^ simulation points in the *x*- and *z*-directions for the photolysis laser (left panel) and the ps-MPI laser (right panel). The area within the white rectangles indicates those trajectories that pass through the experimental apertures. Because of the photolysis beam diameter of 24 mm, the Rayleigh length of the photolysis laser and therefore the effective size of the photolysis volume in the *z*-direction are rather small. Ideally, the photolysis laser should form a line focus in the *x*-direction. However, the change of focal length as a function of photolysis wavelength causes a tilt in the *z*-direction. For the ps-MPI laser operated at 355 nm, we found a linear dependence of the signal intensity on laser pulse energy. The right panel of Figure 5 shows that the ionization volume has a size of ∼4 mm, while the experimental apertures limit the detection to ±1.8 mm.

**Figure 6:**
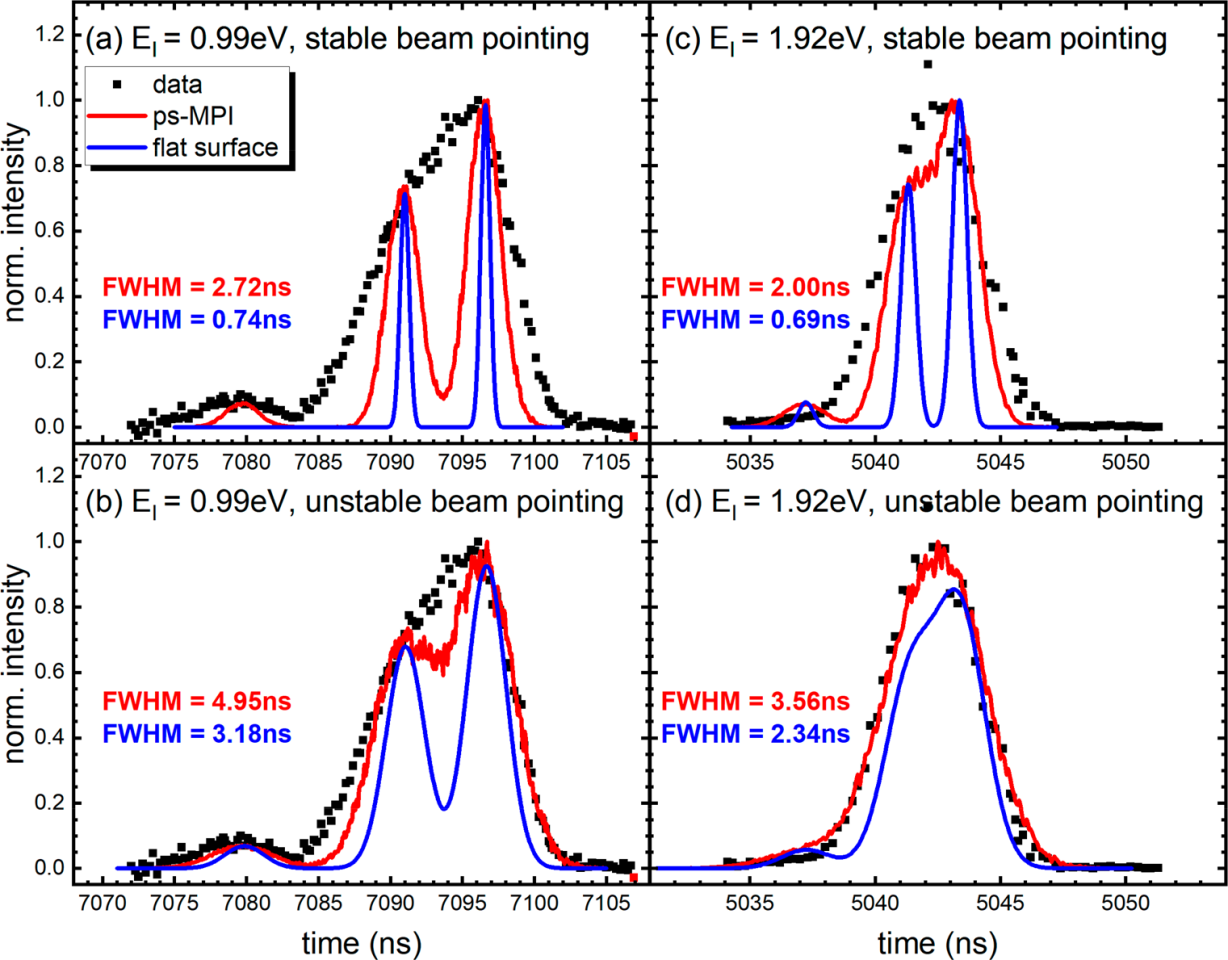
Simulating experimental pulse profiles. Experimentally observed pulses (black squares) are compared to numerical simulations (see text) for *E*_I_ = 0.99 eV (a, b) and 1.92 eV (c, d). Panels a and c show predicted pulses convoluted over the spatial extent of the lasers (Figure 5) assuming perfect pointing stability (red curves) as well as simulated pulses assuming a planar detector (blue curves). Panels b and d show predicted pulses convoluted over the spatial extent of the lasers (Figure 5) with an additional convolution over measured pointing instability (35 μrad standard deviation) of the two laser beams as well as simulated pulses assuming a planar detector (blue curves).

The results of the numerical simulation of the experiment (red lines) are compared to the experimental data (black squares) in Figure 6a for *E*_I_ = 0.99 eV and Figure 6c for *E*_I_ = 1.92. For both incidence energies, it is clear that the simulations predict narrower time distributions than observed experimentally. We also performed simulations assuming a tighter focus of the ps-MPI laser by increasing the initial beam diameter by a factor of 2. As expected, the simulated pulse duration decreases significantly; however, experiments using a more tightly focused laser beam for ps-MPI did not affect the measured H atom pulse duration. These observations suggest that the focal properties of the laser beams are not the only factors influencing the temporal resolution of the observed H atom pulses.

We next measured the pointing stability of the two laser beams using quadrant photodiode detectors and found substantial instability (50–100 μm FWHM over 30 min). Note that to observe a 1 ns pulse for H atoms with 1.92 eV translational energy, the relative pointing stability of the two laser foci must be better than 20 μm. This motivated us to improve our simulations, taking into account the laser beam pointing variations. This was done by adding an angular perturbation to both laser beams in the simulations described above and exploiting eq 10, which provides the relation between the focal position *x* and the incidence angle onto the focusing optics γ.
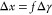
10We assumed a normally distributed random value of γ with a standard deviation of 40 μrad (FWHM = 94 μrad). The simulated pulses including pointing instability are shown as red lines in Figure 6b,d. They agree much better with the experimental observations; indeed, they agree almost perfectly for the 1.92 eV H atom pulses. For the *E*_I_ = 0.99 eV pulses, the simulation also agrees well on the falling edge (long flight times) of the pulse, but the agreement is significantly worse at the rising edge (shorter flight times). We believe this indicates that the bunch compression is not perfectly optimized for this energy. The fact that the simulation gives a good representation of experiment argues that the observed H atom pulse widths are limited by the finite size of the focus of the ps-MPI laser and the pointing stabilities of the photolysis and detection laser beams. If we remove these factors from the simulation, we can simulate the time distribution for the H atoms arriving at a planar surface. This is shown as blue lines in Figure 6a,c. The derived pulse with is ∼700 ps.

**Figure 7:**
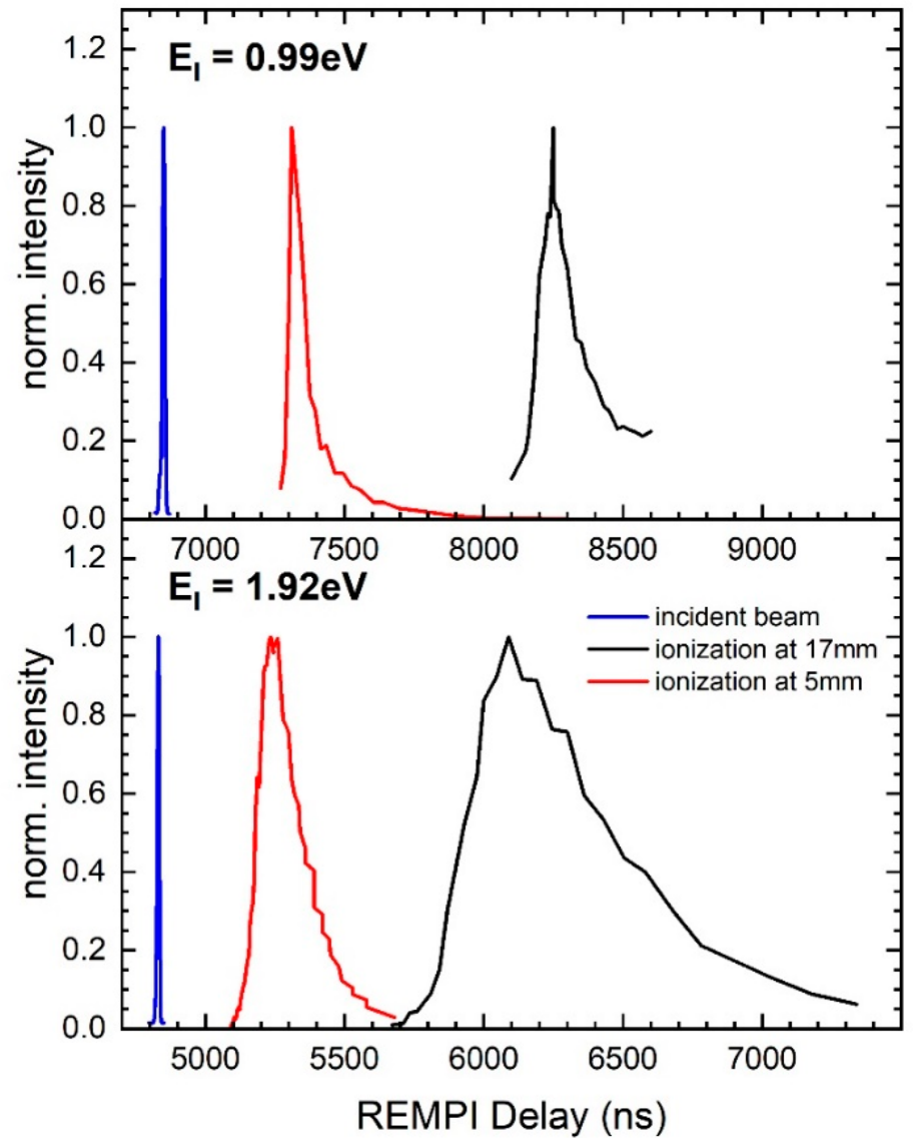
Time profiles of scattered H atoms from EG/Ir(111). The red curves show the scattered time profile for ionization at 5 mm from the surface. The black curves show the profiles for ionization 17 mm from the surface. Incident H atoms are shown as blue curves. All time traces are normalized to the peak for comparison.

We next show that these beams are intense enough to deliver high quality data for H atom scattering from a single crystal of epitaxial graphene (EG) grown on Ir(111). Here we used ns REMPI detection due to its higher sensitivity and the ease of varying the time of arrival of the light pulse compared to experiments with ps-MPI. Figure 7 shows a comparison of time profile for incident H atoms (blue) as well as scattered H atoms detected 5 mm (red) and 17 mm (black) from the surface. The incident H atoms appear as a narrow pulse (shown also in Figure 4a,c). The scattered H atoms appear 5–6 μs later and are broadened due to energy transfer to the surface. The increased broadening with increased flight distance clearly shows the velocity spread in the scattered atoms.

**Figure 8:**
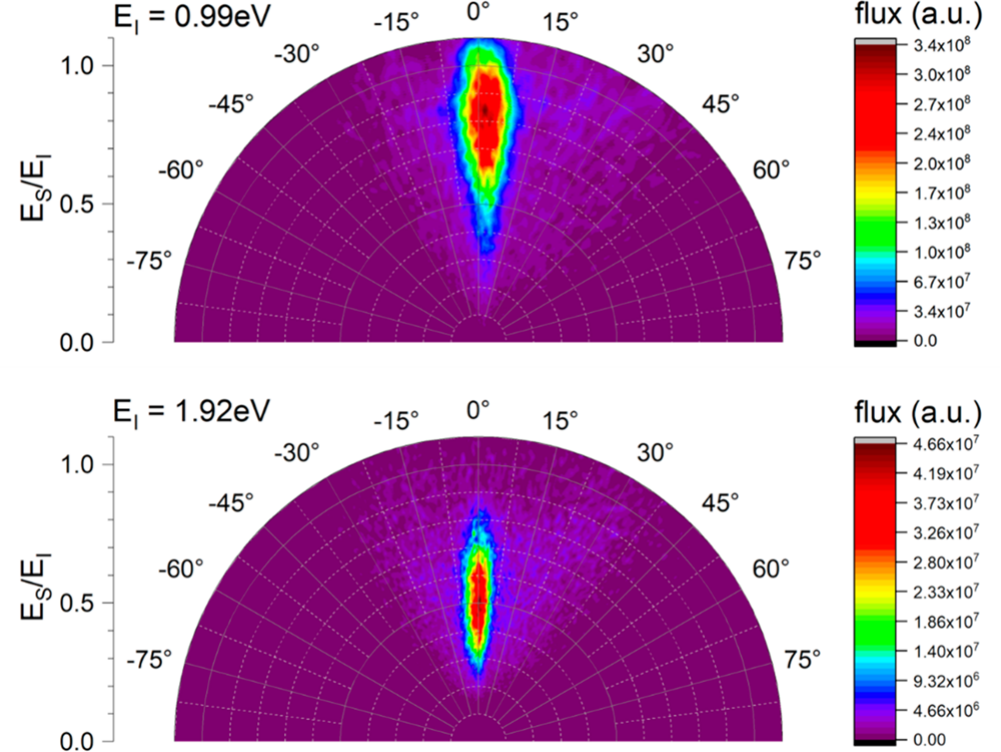
Angular distribution of H atoms scattering off single-crystal EG/Ir(111) for *E*_i_ = 0.99 eV (upper panel) and *E*_i_ = 1.92 eV (lower panel). The color coding reflects the flux observed in the experiment. The REMPI laser focus was 5 mm from the surface (red curves in Figure 7).

At each delay shown in Figure 7, we also obtain velocity map images. By summing these images over all delays, we obtain a composite ion image representing all scattered H atoms. For each pixel in the image, we can assign a velocity component parallel and perpendicular to the surface, from which we can compute the flux and the scattering angle and translational energy. This is shown in Figure 8 as polar plots for both incidence energies.

**Figure 9:**
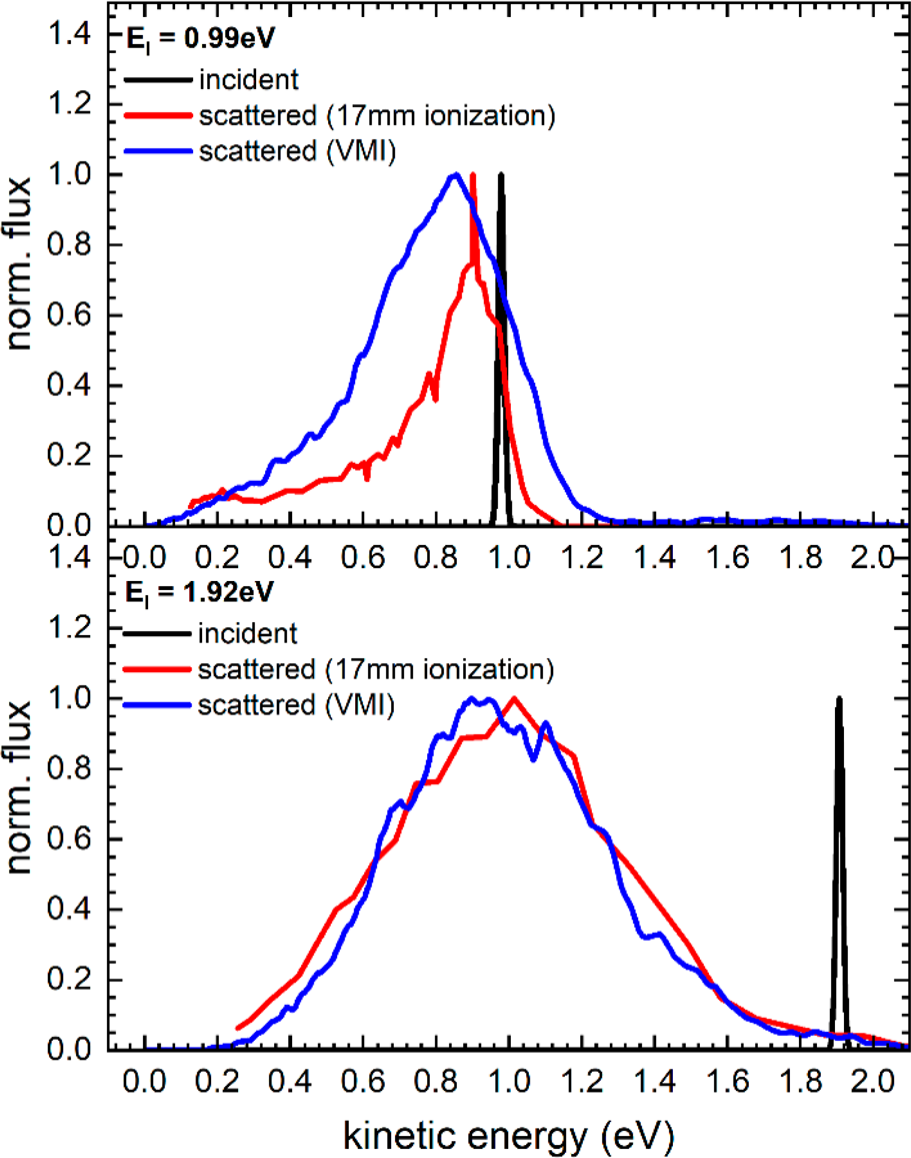
Energy distributions of H atoms scattered from graphene/Ir(111) at *E*_I_ = 0.99 eV (top) and *E*_I_ = 1.92 eV (bottom). The black lines indicate the incident H atom beam. Scattered H atoms are shown as red and blue lines. Red lines indicate energy distributions obtained by positioning the REMPI detection laser 17 mm away from the surface and varying its delay. Blue lines are energy distributions derived from the polar plots Figure 8, where a laser surface distance of 5 mm was employed for velocity map imaging.

Figure 9 shows H atom scattering energy distributions derived from experiments at both incidence energies. The blue curves show the energy distributions obtained from Figure 8 when integrating over all scattering angles. The red curves show the energy distributions extracted directly by scanning the delay between the two lasers with the REMPI laser beam 17 mm from the surface (black curves of Figure 7). For *E*_i_ = 0.99 eV, we see that the blue and red curves deviate from one another—the delay scan results do not report velocities of all scattering angles. Because of the very narrow angular distribution seen for the scattering data at *E*_i_ = 1.92 eV, the two curves agree well. Unless stated otherwise, we consider below only the energy distributions obtained from the 5 mm distance REMPI data employing velocity map imaging. For *E*_i_ = 0.99 eV, the most probable scattering energy is ∼85% of the incidence energy, whereas H atoms impinging at *E*_i_ = 1.92 eV loose on average about half of their translational energy. In both cases there is only one feature.

## 4 Discussion

The results presented above demonstrate the use of short H atoms pulses for obtaining high quality scattering data from graphene. Furthermore, the results are qualitatively similar to prior work of ref (8)—a quasi-elastic feature is seen at low incidence energy, and a large energy-loss channel is seen at high incidence energy. These qualitative similarities are not surprising. In ref (8), H was scattered from polycrystalline graphene grown on Pt(111), whereas the present experiments were performed on single crystal EG grown on Ir(111). The fact that changing the metal substrate does not have a strong influence on the scattering dynamics is consistent with the fact that in both cases graphene interacts with the metal through weak physisorption forces. In fact, molecular dynamics simulations assuming free-standing graphene were quite successful in describing the scattering distributions of the prior experiments.^8,9^

An important difference concerns the experimental geometries of the two studies. This makes direct comparisons impossible and introduces differences between the two experiments that are not fundamental in nature. In the present paper, experiments are only possible for H atoms incident along the surface normal (ϑ_*I*_ = 0°), whereas the work of ref (8) varied ϑ_*I*_ from 30° to 60°. Figure 8 reveals observational differences with the prior work. At *E*_i_ = 1.92 eV, the scattering energy distribution shows a single feature that has a FWHM of about 0.9 eV with an average energy loss of close to 50%. In ref (8) two scattering channels were seen—at low normal incidence energy, quasi-elastic scattering was seen, whereas a strongly inelastic scattering channel grew with increasing normal incidence energy. This was explained by the promotion of chemisorption barrier crossing with increasing normal incidence energy. In the current experiments at *E*_i_ = 1.92 eV and (ϑ_*I*_ = 0°), there is no sign remaining of the quasi-elastic channel. This may, however, be consistent with ref (8), which estimated the sticking probability to be ∼10% at *E*_i_ = 1.92 eV and ϑ_*I*_ = 0°. If correct, it would suggest that impinging H atoms not only have enough kinetic energy to overcome the adsorption barrier but also maintain enough kinetic energy to escape the binding well and scatter back into the gas phase, thus dominating the flux of scattered atoms.

This work shows much narrower scattering angular distributions at both incidence energies (compare Figure 2 of ref (8) to Figure 8). In the prior study, the impinging H atoms have a significant incidence momentum parallel to the surface. Hence, quasi-elastic deflection seen at *E*_i_ = 0.99 eV led to nearly specular scattering, reflecting small potential surface corrugation for trajectories that do not cross the chemisorption barrier. Note that at this incidence energy H atoms that cross the barrier are unlikely to recross. Instead, they stick. But the low-energy sticking occurs where the barrier is lowest, directly atop a C atom. For H atoms that strike the middle of the C six-ring, the barrier is much larger and adsorption is unlikely. It is possible that under the current conditions (*E*_i_ = 0.99 eV, ϑ_*I*_ = 0°) scattered atoms result selectively from H scattering near the middle of the C six-ring, which might produce very narrow angular distributions. Turning to the transient bond formation channel seen in the prior work at *E*_i_ = 1.92 eV and ϑ_*I*_ = 30°–60°, in this case a strong force is present directing the H atom toward the surface normal. This effect explains the relatively broad angular distributions seen in ref (8). In this study employing normal incidence, the transient bond formation directs the outgoing H atom along the surface normal. Hence, there may be a much smaller force inducing momentum parallel to the surface, and again, narrow angular distributions result. In summary, when comparing the two studies, we find differences, but they are subtle. It will be interesting to see if careful comparison to theoretical simulations can reveal the source of the small differences. The fact that the differences are small gives us confidence that the new approach to H atom scattering employing bunch compression and ion imaging does not introduce large artifacts.

## 5 Conclusion

In this work, we have demonstrated the production of short H atom pulses with sufficient intensity to allow for surface scattering experiments. The observed pulse duration using ps-MPI detection is 3.3 ns, but numerical simulations showing that the observable pulse duration is limited by the focal spot size of the ps-MPI detection laser as well intrinsic beam pointing instabilities of photolysis as well as detection laser beams suggest that the true pulse duration is ∼700 ps. In the future, we plan to implement a laser beam stabilization system that can compensate for beam movement on an ∼500 ms time scale limited by the 50 Hz sampling rate. In addition, we plan to establish pulse duration measurements that acquire single shot ion images with synchronized position detection of photolysis and ps-MPI laser beam to obtain the best possible temporal resolution. Future steps will involve H atom scattering with synchronized laser excitation of the surface, i.e., rapid laser heating.^28−31^

We also highlight the fact that bunch compression photolysis is useful for experiments employing conventional excimer lasers for photolytic H atom beam production. By spatially chirping the nanosecond excimer, it is possible to achieve bunch compression, reducing the typical 100 ns long pulses seen in these experiments to about 10 ns. This has the potential to increase signal levels by about a factor of 10×.
